# Treatment of Fraley's syndrome by upper-pole nephrectomy

**DOI:** 10.1590/S1516-31802007000600010

**Published:** 2007-11-01

**Authors:** Thaís Bandeira Cerqueira, Natalia Bacellar Costa Lima, Romeu Magno Baptista Neto, José Cohim Moreira Filho, Luiz Eduardo Café

**Affiliations:** Escola Bahiana de Medicina e Saúde Pública (EBMSP) and Santa Casa de Misericórdia da Bahia, Salvador, Bahia, Brazil

**Keywords:** Nephrectomy, Kidney diseases, Kidney calculi, Hydronephrosis, Low back pain, Nefrectomia, Nefropatias, Cálculos renais, Hidronefrose, Dor lombar

## Abstract

**CONTEXT:**

Fraley's syndrome is characterized by vascular compression on the superior infundibulum with secondary dilatation of the upper pole calyx, mostly located on the right side.

**CASE REPORT:**

We present the case of a 22-year-old woman with vascular compression of the upper-pole infundibulocalyceal system (Fraley's syndrome). The patient had a history of frequent hospitalizations for emergency care due to lumbar pain over the past twelve months. The diagnosis was obtained following renal arteriography. Since the surgical treatment by means of upper-pole nephrectomy, the patient has not had any further symptoms.

## RESUMEN

**CONTEXTO:**

A síndrome de Fraley é caracterizada por impressão vascular do infundíbulo superior com dilatação secundária do pólo caliceal superior. É geralmente localizada no lado direito.

**RELATO DE CASO:**

Nós apresentamos o caso de uma mulher com 22 anos de idade com compressão vascular do sistema infundibulocaliceal do pólo superior (síndrome de Fraley). A paciente trazia história de hospitalizações freqüentes em emergências nos últimos 12 meses devido a cólicas renais. O diagnóstico foi obtido após arteriografia renal. Depois do tratamento cirúrgico por meio de nefrectomia polar superior, a paciente não apresentou mais sintomas.

## INTRODUCTION

Fraley's syndrome is a disease that affects the renal calyces and was first described in 1966 by Elwin E. Fraley.^1^ It consists of an upper-pole infundibulocalyceal obstruction caused by extrinsic intrarenal vessel compression and is mainly characterized by nephralgia and calyectasis. It most frequently affects women and the right kidney.^2,3^ Fraley's syndrome often requires surgical treatment.

The purpose of this report was to describe the case of a patient presenting a history of right flank pain treated by upper-pole nephrectomy. Intrinsic obstruction of the infundibulum was ruled out and Fraley's syndrome was diagnosed by means of arteriography.^1^

## CASE REPORT

This case involved a 22-year-old woman with a history of two years of right flank pain without hematuria, who had required emergency care six times over the past 12 months. During this period, several abdominal ultrasounds and helical computed tomography scans without contrast had been performed. Intravenous pyelography revealed upper calyx dilatation of the right kidney associated with infundibular filling defects, and renal arteriography confirmed the presence of right calyceal obstruction caused by extrinsic compression of the upper branch of the right renal artery.

**Figure 1:**
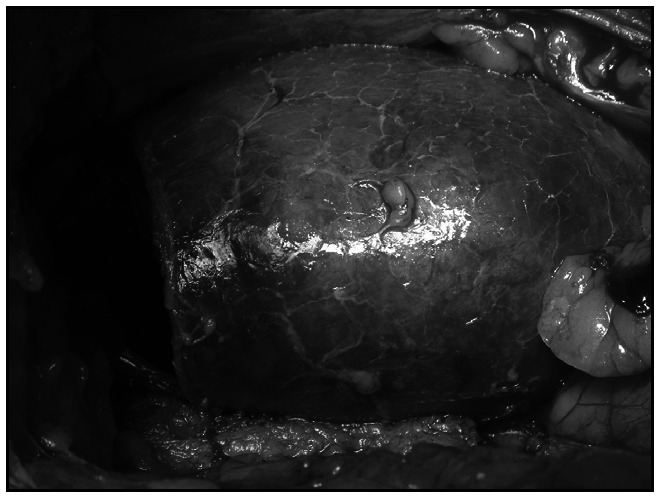
Right renal surgery on a 22-year-old woman with Fraley's syndrome that caused right flank pain. The surgery was carried out by means of lumbotomy, kidney hypothermia, prior clamping of the renal artery and upper-pole nephrectomy.

Surgical treatment was carried out by means of lumbotomy, kidney hypothermia, prior clamping of the renal artery and upper-pole nephrectomy (Figure 1). There were no microscopic alterations to the involved vessel wall, renal parenchyma or dilated calyx, in the surgical specimens. The patient remained free of pain when last seen, 18 months after the operation.

**Figure 2:**
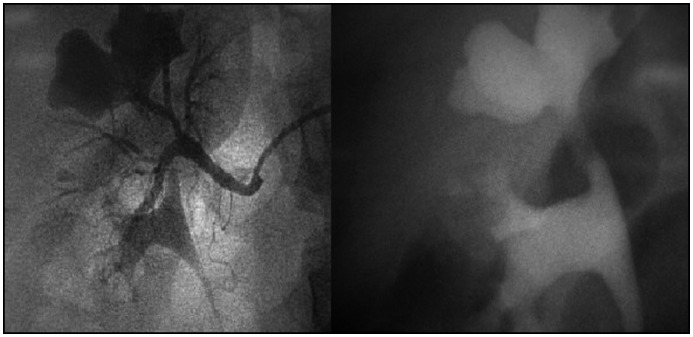
Renal arteriography (left). Excretory urogram with contrast (right). The images show vascular obstruction of the right upper infundibulum by a branch of the renal artery, with secondary dilatation of the upper-pole calyx.

## DISCUSSION

Intrarenal vessels produce infundibular obstruction by compressing the infundibulum against other vessels or the overlying kidney.^1^ In all cases in which the infundibulum is pinched between an arterial branch and a vein, obstruction is clearly due to arterial compression.^1,2^ The exact mechanism through which these vessels cause infundibular obstruction is unknown.^1^

The main clinical manifestation of Fraley's syndrome is flank pain, which is often colicky. Microscopic hematuria occurs less commonly, and the definitive diagnosis is usually delayed because of initial misdiagnosing. Renal colic due to urinary microlithiasis is a frequent initial diagnosis. In this case, the patient had been treated several times in the emergency room with the clinical suspicion of renal colic due to migration of renal calculi. In situations such as this, Fraley's syndrome must be considered and the urinary tract should be evaluated by means of contrast examination. Vascular impressions on the collecting system are frequently viewed while performing an excretory urogram (Fi­gure 2), and these are more common on the right side.^3^ In the present case, the definitive diagnosis was obtained by means of renal arteriography, which is often pathognomonic. Renal ultrasound is very useful, in order to monitor the behavior of the syndrome over time and any upper-pole dilation.^2^

Surgical treatment of Fraley's syndrome is indicated only in symptomatic or complicated cases.^2^ Several surgical techniques have been used, and the choice depends on the extent and severity of the lesion, the type of obstruction and the topographic anatomy of the intrarenal structures.^4^ Because of the infrequency of reports in the literature, it is not possible to define a standard treatment for Fraley's syndrome, and therefore each case should be individualized and it should be sought to preserve as much of the renal parenchyma as possible.^4,5^ Upper-pole partial nephrectomy is often advocated, and this was the option selected in the present case. Other treatment options that have been described include percutaneous placement of a permanent intra-infundibular prosthesis and nephron-sparing procedures using resection of a branch of the renal vein.^4,5^

## CONCLUSION

Infundibular obstruction caused by vascular compression gives rise to symptoms such as unexplained lumbar pain. In such cases, Fraley's syndrome should be investigated. Renal arteriography can provide a definitive diagnosis. Surgical treatment is the best option for symptomatic patients, but there is no standard surgical technique. Partial nephrectomy was performed successfully in our case.
